# Targeting Reactive Oxygen Species in Cancer via Chinese Herbal Medicine

**DOI:** 10.1155/2019/9240426

**Published:** 2019-09-10

**Authors:** Qiaohong Qian, Wanqing Chen, Yajuan Cao, Qi Cao, Yajing Cui, Yan Li, Jianchun Wu

**Affiliations:** ^1^Department of Integrated Traditional Chinese and Western Medicine, Obstetrics and Gynecology Hospital, Fudan University, Shanghai 200011, China; ^2^Department of Oncology, Shanghai Municipal Hospital of Traditional Chinese Medicine, Shanghai University of Traditional Chinese Medicine, Shanghai 200071, China

## Abstract

Recently, reactive oxygen species (ROS), a class of highly bioactive molecules, have been extensively studied in cancers. Cancer cells typically exhibit higher levels of basal ROS than normal cells, primarily due to their increased metabolism, oncogene activation, and mitochondrial dysfunction. This moderate increase in ROS levels facilitates cancer initiation, development, and progression; however, excessive ROS concentrations can lead to various types of cell death. Therefore, therapeutic strategies that either increase intracellular ROS to toxic levels or, conversely, decrease the levels of ROS may be effective in treating cancers via ROS regulation. Chinese herbal medicine (CHM) is a major type of natural medicine and has greatly contributed to human health. CHMs have been increasingly used for adjuvant clinical treatment of tumors. Although their mechanism of action is unclear, CHMs can execute a variety of anticancer effects by regulating intracellular ROS. In this review, we summarize the dual roles of ROS in cancers, present a comprehensive analysis of and update the role of CHM—especially its active compounds and ingredients—in the prevention and treatment of cancers via ROS regulation and emphasize precautions and strategies for the use of CHM in future research and clinical trials.

## 1. Introduction

Reactive oxygen species (ROS) and the oxidative stress that they produce have historically been considered mutagenic and carcinogenic because they can damage macromolecules such as DNA, lipids, and proteins, leading to genomic instability and changes in cell growth [1, 2]. Thus, ROS can contribute to malignant transformation and drive tumor initiation, development, and progression. Therefore, antioxidants are usually thought to be beneficial for both the prevention and treatment of cancer because they can quench ROS and reduce oxidative stress [1]. However, many clinical studies have shown that antioxidant supplements do not reduce the risk of cancer or prevent tumor growth, sometimes even exerting the opposite effects [3, 4]. Then, the protumorigenic effect of antioxidants, as well as their promotion of tumor distant metastasis, was confirmed in mouse models of cancer [5, 6]. This finding emphasized the positive role of ROS in tumor inhibition from the opposite perspective. In this context, the biological functions of ROS in cancer are rather contradictory and ambiguous [7]. As two-faced molecules, ROS not only are associated with deleterious effects but are also signaling molecules involved in multiple cellular signaling pathways important for the fate of both normal and tumor cells [8]. Thus, developing approaches for the rational use of ROS in antitumor applications is very challenging but worthwhile.

Chinese herbal medicine (CHM) has been used in China for approximately three thousand years and has contributed greatly to human health. In addition, as the main components of natural products, CHM has been regarded as an important source for novel lead compounds for the discovery of modern drugs, including anticancer drugs [9]. Currently, an increasing number of cancer patients are using CHM and its derivatives as complementary and alternative drugs; indeed, these medicines display synergistic effects when combined with conventional chemotherapy, radiation therapy, and even molecular targeted agents. Moreover, some have been suggested to have distinctive advantages in treating certain tumors [10]. A few clinical studies have reported that CHMs can alleviate the symptoms of diseases, improve the quality of life, and prolong the survival of cancer patients [11, 12]. However, the underlying mechanisms remain largely unknown. Many active compounds and ingredients in CHM can exert multiple antitumor effects accompanied by changes in cellular ROS. In this article, we comprehensively reviewed the dual roles of ROS in cancers and the ROS-mediated roles of CHM in cancer progression and treatment.

## 2. Generation and Biological Functions of ROS

### 2.1. Generation of ROS

**Figure 1:**
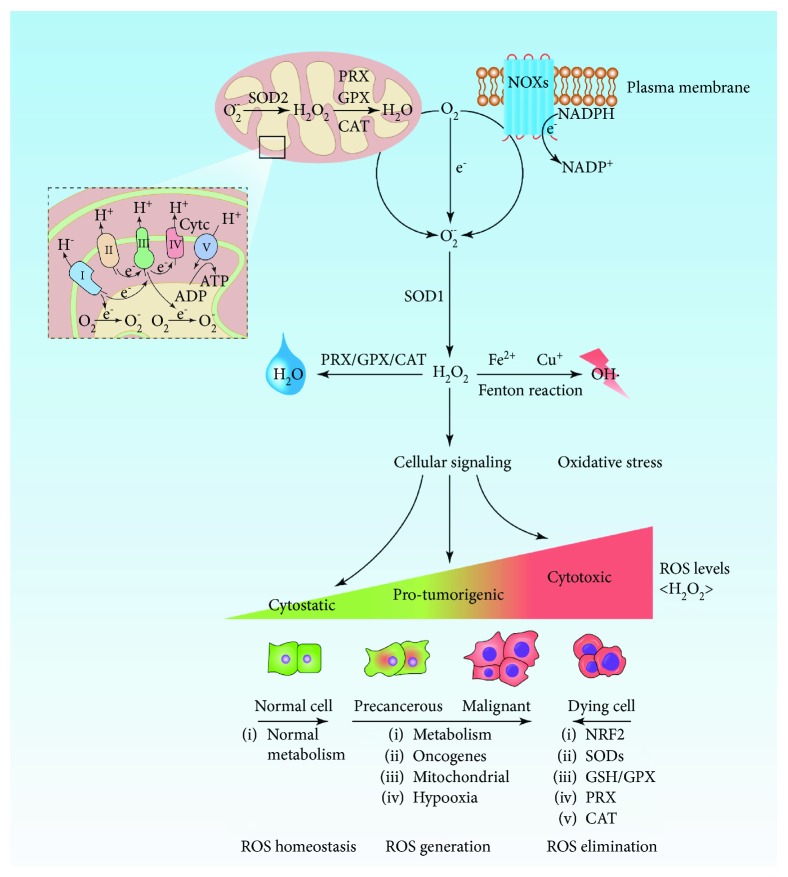
Production, regulation, and biological effects of ROS. Mitochondria and NOXs are the main sources of O_2_^·-^. O_2_^·-^ is formed by molecular oxygen that receives one single electron leaking from mitochondrial ETC or from NOXs. O_2_^·-^ is then rapidly converted into H_2_O_2_ by the corresponding SODs. H_2_O_2_ can be converted into H_2_O through intracellular antioxidants such as PRX, GPX, and CAT. When the H_2_O_2_ level is uncontrollably increased, OH^·^ is further formed via the Fenton reaction with metal ions, thereby damaging biological macromolecules such as DNA, lipids, and proteins. In addition, H_2_O_2_ is a major signaling molecule participating in cellular physiological and pathological processes. The effects of ROS depend on their intracellular concentration. Normal cells typically have lower concentrations of ROS due to their normal metabolism; in normal cells, ROS act as signaling molecules to maintain homeostasis, such as by limiting cellular proliferation, differentiation, and survival. The increased metabolic activity of cancer cells produces high concentrations of ROS, leading to a series of tumor-promoting events, such as DNA damage, genomic instability, oncogene activation, sustained proliferation, and survival. Elevated ROS concentrations also result in the protective growth of cancer cells with enhanced antioxidant capacity to maintain tumor-promoting signaling. Increasing ROS levels to the toxicity threshold, such as by treatment with exogenous ROS inducers or antioxidant inhibitors, causes oxidative damage to cells and, inevitably, cell death.

ROS are broadly defined as oxygen-containing chemical molecules with highly reactive properties and mainly include superoxide anions (O_2_^·-^), hydrogen peroxide (H_2_O_2_), and hydroxyl radicals (OH^·^) [8, 13]. These molecules are by-products of aerobic metabolism and are mainly derived from mitochondria, peroxisomes, and the endoplasmic reticulum (ER), among which mitochondria are the major source—approximately 2% of the oxygen consumed by mitochondria is used to form the superoxide anion [14, 15]. In the process of mitochondrial oxidative phosphorylation, electrons leaking from the electron transport chain (ETC) may react with molecular oxygen to produce O_2_^·-^, a reaction that is primarily mediated by coenzyme Q, ubiquinone, and respiratory complexes I, II, and III [16]. O_2_^·-^ is the precursor form of most other ROS species which can be rapidly converted to H_2_O_2_ by the corresponding superoxide dismutase (SOD). Further, H_2_O_2_ can be converted to OH^·^ by Fenton chemical reactions in the presence of a metal (iron or copper) (Figure 1). In addition to mitochondria, NADPH oxidases (NOXs) are another prominent source of superoxide that can catalyze the formation of O_2_^·-^ from O_2_ and NADPH (Figure 1). Besides, ROS are formed in the cytoplasm by enzymatic reactions involving peroxisomes, xanthine oxidase, cytochrome P450, lipoxygenases (LOXs), and cyclooxygenases.

Intracellular ROS levels are tightly controlled via diverse, complex synthesis and degradation pathways; this tight control is crucial for cellular homeostasis (Figure 1). The ROS-detoxifying system mainly comprises both enzymatic and nonenzymatic antioxidants [7, 17]. Enzymatic antioxidants include SOD, catalase (CAT), glutathione peroxidase (GPX), peroxiredoxin (PRX), and thioredoxin (TRX); nonenzymatic antioxidants include glutathione (GSH), flavonoids, and vitamins A, C, and E [18]. As described above, SOD can rapidly catalyze the conversion of O2^·-^ to H_2_O_2_, which can be further converted to water by the PRX system, the GPX system, and CAT. SOD has three isoforms in mammals: cytoplasmic Cu/ZnSOD (SOD1), mitochondrial MnSOD (SOD2), and extracellular Cu/ZnSOD (SOD3), all of which require specific catalytic metals (Cu or Mn) for activation [19]. PRXs are considered ideal H_2_O_2_ scavengers due to their abundant expression and broad distribution in cellular compartments such as the cytosol, the ER, mitochondria, and peroxisomes. During the metabolism of H_2_O_2_, PRX is oxidized and subsequently reduced by TRX, which is then reduced by thioredoxin reductase (TrxR) via the transfer of electrons from NADPH [20]. In addition to PRXs, GPXs are important scavengers. GPX catalyzes the reduction of H_2_O_2_, leading to the oxidation of GSH to glutathione disulfide (GSSG) that can be reduced back to GSH by glutathione reductase (GR) with NADPH as an electron donor [21].

In addition to antioxidant enzymes, the transcription factor nuclear factor erythrocyte 2-related factor 2 (Nrf2) plays a vital role in regulating the intracellular redox status [17]. Under physiological conditions, Nrf2 is located in the cytoplasm and remains at a low level under the control of Kelch-like ECH-associated protein 1 (KEAP-1). KEAP binds and specifically degrades Nrf2 via the ubiquitin-proteasome pathway. Under oxidative stress, Nrf2 dissociates from KEAP and is translocated to the nucleus. Then, activated antioxidant response elements (AREs), such as GSH, TRX, and PRX, decrease the intracellular ROS levels and protect against cell death [22] (Figure 1).

### 2.2. Biological Functions of ROS

A canonical mechanism by which ROS participate in the regulation of redox signaling is through the oxidative modification of cysteine residues in proteins [16]. During the redox process, reactive cysteine thiol (Cys-SH) can be oxidized by H_2_O_2_ to reversible sulfenic acids (Cys-SOH), resulting in allosteric and functional changes within the protein [8]. This process is reversible; Cys-SOH can be reduced to its original state and restored its function by the TRX and GRX [8, 18]. Meanwhile, Cys-SOH can be further oxidized by continuously elevated ROS to form irreversible oxidation products, such as sulfinic or sulfonic species, causing permanent oxidative damage to proteins. This accounts for the double-sided nature of ROS and to a large extent, depending on its intracellular concentration and duration of exposure.

ROS involve a series of biological effects that are concentration-dependent. At low to moderate levels, ROS function as a second messenger and are involved in mediating cell proliferation and differentiation and the activation of stress-responsive survival pathways by regulating various cytokine receptors, serine/threonine kinase receptors, and G protein-coupled receptors [23, 24]. In contrast, due to their strong oxidizing capacity, ROS at a high level can react with intracellular macromolecules such as phospholipids, nucleic acids, and proteins to produce cytotoxicity. ROS have been linked to many diseases, such as cancers and diabetes [25]. The tight modulation of both ROS-producing pathways and ROS-detoxifying pathways may be required for the control of these diseases [26].

## 3. ROS and Cancer

The dual properties of ROS described above are simultaneously utilized by normal cells and cancer cells to support cell growth and survival. However, most cancer cells have higher levels of ROS than normal cells due to their enhanced glucose metabolism (the Warburg effect), mitochondrial dysfunction, and oncogenic activity [18, 27]. On one hand, this property enables the activation of central protumorigenic signaling pathways. On the other hand, the resulting oxidative stress may also exert potential antitumor effects [8]. In the next sections, we discuss how ROS can either promote or inhibit cancer progression, providing the clues for anticancer therapies based on redox regulation.

### 3.1. Pros of ROS in Cancer

Moderately increased levels of ROS are a pivotal driving factor of tumor initiation, development, and progression [24, 26] (Figure 1). In the initial stage of tumor formation, ROS may function as a direct DNA mutagen, induce genomic instability, damage mitochondrial DNA, and activate various signaling cascades to trigger malignant transformation [28–31]. In addition to causing significant genetic changes, ROS may alter the expression of oncogenes or tumor suppressor genes by mediating epigenetic modifications such as methylation or acetylation, thereby promoting carcinogenesis [32]. Conversely, these events may, in turn, promote ROS production and accumulation, leading to further oxidative DNA damage and the malignant deterioration of cells to aid tumor formation [28, 32].

Existing tumors exhibit several noticeable characteristics, including sustained proliferation, apoptosis resistance, angiogenesis, invasion and metastasis, and tumor-promoting inflammation [33]. ROS are involved in all of these processes, which are conducive to tumor survival and development. ROS regulate the activities of many proteins and signaling pathways, thereby facilitating tumor cell proliferation and death evasion. For example, ROS can transiently inactivate tumor suppressors such as phosphatase and tensin homologue (PTEN) [34], protein tyrosine phosphatases (PTPs) [35], and MAPK phosphatases [36] by oxidative modulation, thereby stimulating the prosurvival PI3K/AKT and MAPK/ERK signaling pathways. Importantly, multiple transcription factors, such as activator protein 1 (AP-1), nuclear factor-*κ*B (NF-*κ*B), Nrf2, and hypoxia-inducible factor-1*α* (HIF-1*α*), which are involved in the control of genes in cell proliferation, are also regulated by increased levels of ROS [37].

Tumor-associated neovasculature formation, or angiogenesis, provides oxygen and nutrients for the continued growth of cancer cells and is a key step in tumor growth and metastasis [38]. A wealth of evidence has shown that ROS play an essential role in tumor angiogenesis through mediating the following events. ROS, especially H_2_O_2_ derived from NOXs, selectively promote endothelial cell (EC) proliferation and survival [39] and prevent apoptosis. Furthermore, ROS-mediated cadherin/catenin phosphorylation leads to the disassembly of EC junctions and promotes cell migration [40, 41]. Additionally, ROS activate VEGF signaling via multiple pathways, including through the induction of the principal regulator HIF-1*α*, which increases VEGF and VEGFR expression [42], and, as mentioned before, through the induction of the PI3K/Akt and MAPK pathways, which activates angiogenic signaling cascades for the upregulation of VEGFR expression. Consistent with this pattern, an increase in the level of extracellular SOD may also suppress the hypoxic accumulation of HIF-1*α* and its downstream target gene VEGF in several different types of cancer cells [43, 44].

Metastasis is a ubiquitous event in cancer development and encompasses a wide array of cellular changes, including the loss of cell-to-cell adhesion, the survival of cells upon matrix detachment, and the ability of cells to migrate and penetrate the basement membrane; ROS are involved in all of these processes [16, 45]. Indeed, ROS generated from NOXs are necessary for invadopodium formation and function in Src-transformed cell lines [46]. Similarly, ROS enable the direct oxidation of the protein tyrosine kinase Src, thereby enhancing the invasive potential, anchorage-independent growth, and survival of Src-transformed cells [47]. Furthermore, H_2_O_2_ has been demonstrated to activate FAK in a PI3 kinase-dependent manner to accelerate cell migration [48]. ROS also participate in the abnormal activation of many proteolytic enzymes, such as MMP, uPA, and cathepsins, facilitating cell migration [49, 50]. Several tumor invasion signaling pathways upstream of MMPs and uPAs, such as the MAPK, PI3K/Akt, and PKC pathways and those modulated by defined transcription factors (AP-1 and NF-*κ*B), are modulated by ROS [50, 51]. Besides, ROS may induce the expression of transcription factors such as Snail and HIF-1*α* [52], leading to epithelial to mesenchymal transition (EMT), an aggressive behavior favoring cancer metastasis and involved in drug resistance[53, 54].

### 3.2. Cons of ROS in Cancer

As stated earlier, cancer cells exhibit higher levels of ROS than normal cells, which contributes to tumor formation and development. However, excessive high levels of ROS can block cell cycle and induce different types of cell death, including apoptosis, autophagic cell death, ferroptosis and necroptosis. Due to space limitations, we focus on the first three types of cell death.

Apoptosis is the most common form of programmed cell death (PCD) in multicellular organisms with typical morphological and biochemical features. Also, apoptosis is a highly regulated process in which cells undergo self-destruction. The two well-known signaling mechanisms are the extrinsic death receptor pathway and the intrinsic mitochondrial pathway. ROS have been demonstrated to be implicated in the activation of both [55, 56]. ROS can activate the transmembrane death receptors such as Fas, TRAIL-R1/2, and TNF-R1 and then recruit the adaptor proteins FADD and procaspase-8/-10 to form death-inducing signaling complexes (DISCs), subsequently triggering the caspase activation and apoptosis [55]. Besides, ROS have been shown to posttranscriptionally inhibit c-FLIP, which suppresses DISC formation, thus causing the activation of the extrinsic apoptosis pathway [57]. Alternatively, ROS may activate ASK1 by oxidizing Trx, resulting in the subsequent induction of apoptosis through MAPKs such as JNK/p38 [58].

In the intrinsic apoptosis pathway, ROS at an elevated level destroy mitochondrial membranes, causing the release of cytochrome c from mitochondria and the induction apoptosis [18, 59]. Cytochrome c forms an apoptotic complex with apoptotic protein activating factor 1 (Apaf-1) and precaspase 9, resulting in the activation of caspase-9, followed by the induction of effector molecules such as caspase-3/7 [60]. The substantial loss of cytochrome c from mitochondria further increases ROS generation due to the disruption of the mitochondrial ETC [55]. Furthermore, ROS have been shown to regulate the activities of both antiapoptotic (Bcl-2, Bcl-X, and Bcl-wl) and proapoptotic (Bad, Bak, Bax, Bid, and Bim) Bcl-2 family proteins, which play an essential role in regulating the intrinsic pathway of apoptosis [61, 62].

In addition, ROS may act as upstream signaling molecules through the ER pathway, another important intrinsic apoptosis pathway. ROS at an excessive level trigger protein misfolding, leading to the unfolded protein response (UPR) and the induction of CHOP, thereby initiating apoptosis by regulating the expression of Bcl-2 family genes [63]. Moreover, ROS can stimulate the release of Ca^2+^ in the ER lumen [64]. Due to the proximity of mitochondria to the ER, when a large amount of Ca^2+^ is released from the ER, a substantial amount of Ca^2+^ is absorbed by mitochondria, causing Ca^2+^ overload in mitochondria. This leads to stimulating the opening of mitochondrial permeability transition pores (MPTPs) leading to the release of ATP and cytochrome c, which further enhance apoptosis and increase ROS generation [65, 66]. Besides, ER stress-mediated apoptosis is partly controlled by the ASK-1/JNK cascade, which is directly regulated by ROS, as mentioned above.

Autophagy (macroautophagy), which is considered a cell survival mechanism to maintain cellular homeostasis, is multistep characterized by the formation of double-membrane autophagosomes by which cells utilize lysosomes to degrade and recycle their damaged organelles and macromolecules [67]. However, depending on the context, autophagy can function as a cell death mechanism and a tumor suppressor mechanism [68]. Many anticancer agents can induce autophagy in cancer cells. Some of them can induce ROS-dependent autophagy leading to cell death (autophagic cell death) [20, 69, 70].

ROS appear to be a key regulator of autophagy under different conditions and are involved in both the protective and toxic effects of autophagy [71]. Currently, several significant mechanisms by which ROS affect autophagy have been revealed. Under starvation conditions, H_2_O_2_ can oxidize and inactivate ATG4, thereby contributing to the increased formation of LC3-associated autophagosomes [72]. ROS may directly trigger the oxidation of ATM to induce AMPK phosphorylation, which inhibits mTORC1 activation and phosphorylates the ULK1 complex to induce autophagy [73–75]. Also, AMPK can be phosphorylated by its upstream kinase AMPK kinase (AMPKK), leading to the induction of autophagy. In an alternative mechanism, H_2_O_2_ activates Bcl-2/E1B interacting protein 3 (BNIP3) to suppress the activity of mTOR and abolish the interaction between Beclin-1 and Bcl-2, causing Beclin-1 release and autophagy induction [76, 77]. Besides, ROS can modulate autophagy by affecting the activity of various transcription factors such as NF-*κ*B, resulting in the expression of autophagy-associated genes (BECN1/ATG6 or SQSTM1/p62) in cancer cells [78, 79].

In contrast, autophagy can reduce ROS levels through the NRF/KEAP1 and P62 pathways [80]. In response to ROS, P62 is activated and thus interacts with KEAP1 to contribute to the suppression of NRF2 degradation and the promotion of its activation, which, in turn, can activate antioxidant defense genes such as GPX, SOD, and TRX [79, 81]. This process contributes to the regulation of autophagy.

Ferroptosis, first named by Dixon et al. in 2012, is emerging as a new form of PCD characterized by the accumulation of cellular ROS in an iron-dependent manner [82, 83]. Ferroptosis is primarily caused by an imbalance in the production and degradation of intracellular lipid ROS and can cause iron-dependent oxidative cell death through a reduction in antioxidant capacity and an accumulation of lipid ROS. Many compounds can induce ferroptosis to kill cancer cells in a manner mainly related to the metabolism of amino acids/GSH, lipids, and iron and the regulation of P53 [84].

Erastin can inhibit the activity of the cysteine-glutamate antiporter (system X_C_^−^), reduce cystine uptake, and lead to the associated depletion of intracellular GSH, in turn causing toxic lipid ROS accumulation and ferroptosis [82, 85]. Inhibition of glutathione peroxidase 4 (GPX4), a GSH-dependent enzyme required for the elimination of lipid ROS, can trigger ferroptosis even at regular cellular cysteine and GSH levels [86]. Other lipophilic antioxidants, such as Trolox, ferrostatin-1, and liproxstatin-1, can inhibit ferroptosis [82, 87]. Intracellular iron is another essential regulator of lipid ROS production and ferroptosis induction. In the presence of iron, lipid hydroperoxides are converted into toxic lipid free radicals, leading to lipid oxidative damage and cell death [88, 89]. Indeed, various iron chelators such as deferoxamine and ciclopirox can abolish ferroptotic cell death caused by system X_C_^−^ inhibitors, GPx4 inhibitors, and GSH depletion [83]. Consistent with this observation, silencing TFRC, thus inhibiting the transport of iron into the cytoplasm, can antagonize erastin-induced ferroptosis [90]. Additionally, PKC-mediated HSPB1 phosphorylation inhibits ferroptosis by reducing the production of iron-dependent lipid ROS, but inhibition of HSF1-HSPB1 pathway activity and HSPB1 phosphorylation increases the anticancer activity of erastin [91]. Together, these results demonstrate the importance of lipid ROS and iron in promoting ferroptosis.

Recent studies have revealed a new mechanism by which P53 acts as a tumor suppressor gene to inhibit tumors by inducing ferroptotic cell death. Jiang et al. demonstrated that P53 could downregulate the expression of SLC7A11, thereby preventing system X_C_^−^ from absorbing cystine, resulting in decreased cystine-dependent GPX activity and cellular antioxidant capacity, in turn leading to ROS-induced ferroptosis and tumor suppression [92]. Indeed, this finding is contrary to those of many other reports showing that P53 can reduce cellular levels of ROS. When ROS levels are low, P53 may prevent the accumulation of ROS from promoting cell survival, whereas when ROS levels are excessive, P53 may evoke cell death via ferroptosis. Currently, P53 is reported to exert a complex and dynamic regulatory effect on ROS, but the role of this regulation in tumors needs further study [93].

## 4. Anticancer Effects of CHM via ROS

As described above, ROS have dual roles in tumor suppression and tumor promotion depending on their concentrations. Moreover, most cancer cells have higher basal levels of ROS than normal cells, which is beneficial for their survival and development. In response to their high basal levels of ROS, the antioxidant capacity of cancer cells is upregulated to maintain redox balance and prevent ROS levels from excessively increasing to induce cell death [8, 26]. However, this effect is very limited in tumor cells. Therefore, either increasing or reducing ROS can be an effective strategy in cancer therapy by disrupting redox balance in tumor cells [17, 20]. CHM has a long history of treating various diseases and is becoming an integral part of comprehensive cancer treatment in China. According to the literature, CHM plays a significant role in cancer therapy through several aspects: reducing inflammatory and infectious complications surrounding the tumors, protecting normal tissues from the possible damage caused by chemo/radiotherapy, enhancing the potency of chemo/radiotherapy and molecular targeted therapies, improving immunity and body resistance to disease, improving general condition and quality of life, and prolonging the survival of advanced cancer patients [94]. However, in most cases, the chemical and pharmacological mechanisms of CHM are ambiguous. The majority of researches on the molecular mechanism of CHM were carried out with an active monomer or crude extract of a single herb, and results indicate that the anticancer activities of diverse CHMs are associated with ROS regulation. Therefore, we summarize current data regarding the ROS-related anticancer effects of CHM on the prevention and therapy of cancers. Some typical Chinese herbal compounds and ingredients are discussed. For additional examples, please see the Tables 1 and 2.

### 4.1. Antioxidant Effects of CHM in Cancer Progression

**Figure 2:**
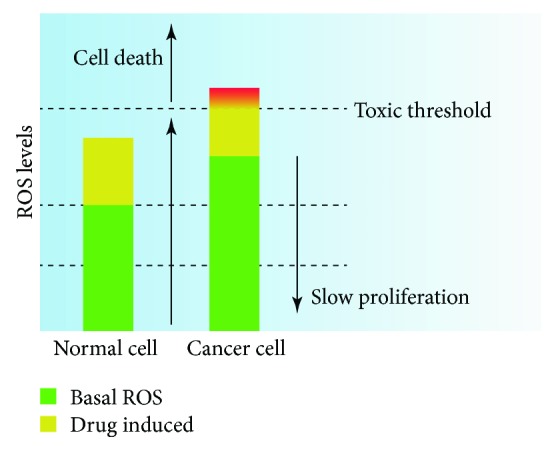
Treatment strategy based on redox regulation. Compared to normal cells, cancer cells have increased levels of basal ROS, resulting in the maintenance of tumor-promoting signaling in these cells. Therefore, strategies for reducing ROS by using antioxidants to prevent carcinogenesis or delay tumor growth are theoretically feasible (however, most current clinical results are not supported). However, strategies for increasing ROS to toxic levels by using ROS inducers and/or inhibiting ROS scavengers can result in the specific killing of cancer cells (such approaches seem more promising than ROS reduction strategies).

Carcinogenesis is a multistep process in which various genetic and epigenetic events occur through the stimulation of numerous inflammatory mediators and ROS production, resulting in the conversion of normal cells into cancer cells [1]. Many carcinogens, such as irradiation, UV light, and toxins, are also exogenous ROS inducers that accelerate the malignant transformation and promote tumor progression by increasing intracellular oxidative damage and activating cancer-promoting signals. Thus, approaches to enhance the antioxidant enzyme system or reduce ROS generation can be used to prevent tumorigenesis and slow tumor progression (Figure 2). There is a beneficial inverse relationship between the consumption of fruits and vegetables and the risk of lung cancer, due to the high antioxidant content of these foods [95, 96]. Increasing types of CHM-derived bioactive ingredients or crude extracts have been shown to suppress chronic inflammation of tissues and prevent carcinogenesis. This effect, to a certain extent, is attributed to the fact that such CHMs are homologous to food and are rich in antioxidants such as saponins, flavonoids, and polysaccharides, which can reduce the oxidative damage caused by excess ROS in normal cells [97].

Studies have shown that the overproduction of ROS induced Cr(VI)-mediated carcinogenesis. Quercetin, one of the most abundant dietary flavonoids in fruits, vegetables, and many CHMs such as *Hippophae fructus* (Sha Ji) and *Lycii fructus* (Gou Qi Zi), has potent antioxidant and chemopreventive properties [98]. Quercetin can protect human normal lung epithelial cells (BEAS-2B) from Cr(VI)-mediated carcinogenesis by targeting miR-21 and PDCD4 signaling, reducing ROS production [98]. Purslane polysaccharides (PPs), a principal bioactive constituent of the *Portulaca oleracea L*. (Ma Chi Xian), possess a wide range of antioxidant, immunomodulatory, and antitumor activities. Methylnitronitrosoguanidine (MNNG) is a carcinogen and mutagen commonly used in experiments. A recent study showed that PPs provide dose-dependent protection against MNNG-mediated oxidative damage by increasing the activity of SOD, CAT, and GSH-Px in gastric cancer rats [99].

During tumor growth, ROS are continuously accumulated by the stimulation of various growth factors and hypoxia-inducing factors in the microenvironment, which in turn accelerates the progression of the tumor and maintain typical hallmarks of cancer. Some CHMs can inhibit tumor growth and progress by reducing ROS production in vitro and in vivo using a mouse model. *Forsythia suspensa* (Lian Qiao), one of the most fundamental medicinal herbs in China, has extensive pharmacological activities and is generally used to treat infectious diseases of the respiratory system. In the past decades, its antineoplastic activity has attracted more attention. *Forsythia fructus* aqueous extract (FAE), as the primary bioactive ingredient of *Forsythia suspensa*, has shown distinct anticancer properties both in vitro and in vivo. FAE can inhibit proliferation and angiogenesis of melanoma cells by antioxidant and anti-inflammatory mechanisms such as in reducing ROS, malondialdehyde (MDA), and IL-6 levels and in increasing GSH, Nrf2, and HO-1 expression [100]. Similarly, andrographolide (AP), a bioactive compound present in the medicinal plant Andrographis paniculata (Chuan Xin Lian), possesses several beneficial properties, including anti-inflammation, antioxidation, and antitumor activities. AP can antagonize TNF-*α*-induced IL-8 release by inhibiting the NOX/ROS/NF-*κ*B and Src/MAPKs/AP-1 signaling pathways, subsequently suppressing angiogenesis in colorectal cancer cells [101]. Isoliquiritin (ISL) is a natural chalcone flavonoid derived from licorice compounds and has antioxidant and antitumor properties. Previous studies have demonstrated that ISL may selectively inhibit prostate cancer cell proliferation by decreasing ROS levels, thus blocking AMPK and ERK signaling [102]; furthermore, this compound can suppress the invasion and metastasis of prostate cancer cells possibly via decreased JNK/AP-1 signaling [103]. Abnormal cell energy metabolism is one of the core hallmarks of cancer [33]. Resveratrol (RSV) is a polyphenolic compound present in many types of fruits, vegetables, and Chinese medical herbs. Numerous studies have shown that it has a variety of biological and pharmacological activities, such as antioxidant, anti-inflammatory, antiaging, and antitumor. RSV can inhibit invasion and migration by suppressing ROS/miR-21-mediated activation and glycolysis in pancreatic stellate cells (PSCs) [104].

In addition, ROS are involved in the antitumor activity of many chemotherapeutic agents, small molecular targeted drugs, and radiation therapy, as well as their side effects [105, 106]. The rational use of the antioxidant effects of CHMs can relieve the toxic side effects of chemo- and radiotherapy on normal cells by eliminating excessive ROS. Sulforaphane is a component of cruciferous vegetables and some Chinese medicinal plants [107]. Studies have shown that sulforaphane is a powerful natural antioxidant to prevent, delay, and improve some side effects of chemotherapy. Sulforaphane can result in the high expression of HO-1 by activating the KEAP1/NRF2/ARE signaling pathway, which protects myocardial cells from doxorubicin-induced oxidative injury and protects the gastric mucosa against *H. pylori*-induced oxidative damage [107, 108]. Ginseng is often used alone or in combination with other herbs for the adjuvant treatment of tumors [109]. Ginsenoside is the main pharmacologically active ingredient of ginseng in exerting anticancer activity. As the primary active component, ginsenoside Rg3 can mitigate doxorubicin-induced cardiotoxicity by ameliorating mitochondrial function, improving calcium handling, and decreasing ROS production [109]. Furthermore, Rg3 inhibits gemcitabine-induced resistance by eliminating ROS, downregulating NF-*κ*B and HIF-1*α*-mediated PTX3 activity [110]. Ginsenoside Rg1 is another ingredient of ginseng and was found to alleviate cisplatin-induced hepatotoxicity via restraining the binding of Keap1 to Nrf2, partly via p62 accumulation, and enhancing Nrf2-related antioxidant activity [111]. *Schisandra sphenanthera* extract has a protective effect against cisplatin-induced nephrotoxicity by activating the Nrf2-mediated defense response, thus increasing GSH levels and reducing ROS levels [112]. Astragalus has a long history of treating immunodeficiency diseases in China and beyond and is often used to reduce side effects caused by chemotherapy [113]. Astragaloside IV (As-IV) is a natural saponin extracted from Astragalus membranaceus, which has antioxidant, anti-inflammatory, and antiapoptotic effects. Studies showed that As-IV markedly ameliorates BLM-induced pulmonary fibrosis in mice, an effect associated with its antagonism of bleomycin-induced oxidative stress and inflammatory responses, increasing SOD activity and total antioxidant capacity in lung tissue and reducing ROS, MDA, and IL-1*β* levels [113].

Notably, some compounds or ingredients of CHM are generally considered to be antioxidants but can induce prooxidant effects similar to those of antioxidant supplements such as vitamin C [114]. These substances exhibit antioxidant activity at low concentrations but induce ROS production and cytotoxicity at high concentrations [115]. For example, the previously mentioned antioxidant ISL initially decreased the levels of ROS in HepG2 cells in a time-dependent manner; along with this effect, the activity of the Nrf2-mediated antioxidant enzyme system also declined to maintain the new redox balance. However, the intracellular ROS level was significantly higher after 6 h of ISL treatment, an effect attributed to reduced antioxidant capacity, and the sensitivity of cancer cells to X-ray irradiation was thus increased [115]. Epigallocatechin gallate (EGCG) is a phenolic compound in green tea extract and has anticancer activities in vivo and in vitro [116]. EGCG can decrease lipid peroxidation in hepatocytes and enhance antioxidant capacity. However, high concentrations of EGCG destroy the mitochondrial membrane and generate intracellular oxidative stress [117]. Thus, whether EGCG exhibits antioxidant or prooxidant activity depends on the cellular stress conditions, cell type, and EGCG concentration [116, 118]. CHM-derived compounds such as quercetin, curcumin, and resveratrol were found to exhibit similar features [3, 119].

To sum up, the antioxidant effects of CHMs described above and the examples listed in Table 1 exhibit diverse anticancer effects, including reducing inflammatory mediators, inhibiting tumor proliferation, inducing antiangiogenesis, suppressing metastasis, inhibiting glycolysis, overcoming drug resistance, and countering the side effects of chemo- and radiotherapy. These effects were mainly achieved by the regulation of several ROS-related transcription factors such as NRF2, NF-*κ*B, COX-2, STAT3, and HIF-1a and by enhancing the capacity of antioxidant enzymes such as GSH, SOD, and HO-1.

### 4.2. Prooxidant Effects of CHM in Cancer Progression

Since the levels of ROS in tumor cells are higher than those in normal cells, tumor cells are potentially more vulnerable to the accumulation of ROS. The strategy of increasing intracellular ROS levels by increasing ROS production and/or inhibiting the antioxidant capacity enables the ROS level to reach the toxic threshold in cancer cells before it does in normal cells, thereby selectively killing tumor cells without causing visible damage to normal cells (Figure 2).

Many CHM compounds can promote the production of intracellular ROS to induce various types of programmed cell death and enhance the efficacy of chemo- and radiotherapy. Scutellaria (Huang Qin) is one of the most commonly used CHMs in China and its surrounding areas and has a practical effect on infectious diseases caused by bacteria and viruses [120]. As a principal bioactive constituent of Scutellaria, wogonin has apparent anticancer effects against different types of cancer cells. It can induce mitochondrial apoptosis by activating PLC*γ*1 via H_2_O_2_ signaling in malignant T cells, resulting in Ca^2+^ overload in mitochondria [120]. Furthermore, wogonin enhanced TRAIL-induced apoptosis through ROS-mediated downregulation of the cFLIPL and IAP proteins [121]. In addition, levistolide A (LA), a natural compound isolated from the Chinese herb *Ligusticum chuanxiong* Hort., can trigger ER stress-induced apoptosis by activating the ROS-mediated PERK/eIF2*α*/CHOP axis [122]. Besides, LA synergizes with vinorelbine against tumors and induces cell cycle G2/M arrest and apoptosis; interestingly, it can reverse P-glycoprotein-mediated multidrug resistance in breast cancer cells [123]. Other classical compounds of CHM that target the apoptotic signaling pathway have been reviewed [9]. Sanguinarine (SNG) is a benzophenanthridine alkaloid that is predominantly extracted from *Chelidonium majus* (Bai Qu Cai), a well-known CHM mainly used for digestive and respiratory inflammatory diseases and malignant tumors. SNG has diverse biological activities, such as antimicrobial, anti-inflammatory, and antitumor properties. Our previous study has shown that SNG successfully inhibited the proliferation of specific lung cancer cells expressing stem cell characteristics, possibly by downregulating WNT/*β*-catenin signaling [124]. SNG can not only induce apoptotic cell death but also trigger autophagic cell death by the ROS-dependent activation of ERK1/2 in malignant glioma cells [125]. Besides, this compound can upregulate NOX3 and then elevate ROS levels, resulting in EGFR^T790M^ degradation to overcome tyrosine kinase inhibitor (TKI) resistance [126]. Artesunate is a derivative of the natural compound artemisinin, which is known for its antimalarial agents, with well-understood pharmacokinetics. ART specifically induces PCD in different cancer types in a manner initiated by ROS generation [127]. Recent studies have found that ART specifically induces ferroptotic cell death in pancreatic cancer cells in a ROS- and iron-dependent manner and that this induction can be blocked by the ferroptosis inhibitor ferrostatin-1 [128]. Interestingly, dihydroartemisinin (DAT), another artemisinin derivative with high bioavailability, enhances the sensitivity of cancer cells to ferroptosis inducers in a lysosome-dependent, but autophagy-independent manner. Importantly, DAT can further improve the ferroptosis-resistant cancer cell lines more sensitive to ferroptotic death, which suggests that the combination of DAT and ferroptosis inducers is an effective anticancer method [129].

In addition to directly inducing ROS production, inhibiting the activity of antioxidant enzymes to increase ROS levels is another potentially more effective approach to kill cancer cells. Cancer cells tend to have higher antioxidant capacity than normal cells to adapt to elevated levels of ROS, which promotes cancer cell resistance to exogenous ROS-inducing agents [119]. Many antioxidants, such as GSH, TRX, and SOD, and Nrf2 activity aid tumorigenesis and confer chemoresistance and are present at high levels in various tumor types [130–133].

Piperlongumine (PL) is a natural constituent of the long pepper fruit (*Piper longum*), which is extensively used in digestive diseases such as gastrointestinal cancer. PL can selectively kill a variety of tumor cells and enhance cisplatin-mediated anticancer activity [134, 135]. Its anticancer effects are mainly attributed to the silencing of the GSTP1 gene, thus reducing GSH content [136]. Isoforretin A (IsoA) is a novel ent-kaurane constituent isolated from a traditional Chinese medicinal herb of the Isodon genus and has multiple anticancer effects both *in vitro* and *in vivo.* IsoA inhibits Trx1 activity by covalently binding to the Cys32/Cys35 residues in the Trx1 activation site, resulting in ROS accumulation and causing DNA damage and apoptosis in tumor cells. It can be a potential novel agent for cancer therapy [137]. Consistent with this effect, both shikonin [138, 139] and parthenolide [140] can inhibit TrxR, interfere with redox balance, and eventually lead to ROS-mediated tumor cell death. Brusatol (BR), the main active ingredient of the *Brucea javanica* plant, has many anticancer properties [141]. BR is a potent inhibitor of Nrf2 and can degrade Nrf2 by ubiquitination to suppress the Nrf2-dependent protective response and thus sensitize lung cancer cells to cisplatin [142]. Moreover, the combination of BR and UVA irradiation increases ROS-induced cell cycle arrest and cellular apoptosis and inhibits melanoma growth by regulating the AKT-Nrf2 pathway in cancer cells [143].

Summarizing the above examples of prooxidant CHM and those listed in Table 2, contrary to the antioxidant effects of CHM, it can be concluded that the prooxidant effects of CHM in cancer cells are achieved by enhancing ROS production and/or inhibiting antioxidant capacity, thereby activating ROS-dependent killing patterns on cancer cells. So far, the killing model of prooxidant CHM is mainly induced by apoptosis, which is primarily achieved by the regulation of ROS-related apoptotic upstream signaling pathway, such as MAPK/JNK/p38, JAK/STAT, PI3K/AKT, and ER stress pathways, followed by activation of apoptotic executive molecules, such as BAX/BCL-2, caspase family, and PARP-1. Other than this, CHMs also induce autophagic cell death, necroptosis, and ferroptosis in uncommon ways, but underlying molecular mechanisms remain unclear. Of note, ferroptosis, as a newly discovered type of cell death, possibly provides a promising choice for the application of CHM in cancer therapy, especially in the case of many conventional agents with apoptosis resistance.

## 5. Discussion

Cancer cells exhibit higher levels of ROS than normal cells [18, 27, 144]. ROS promote tumorigenesis via malignant transformation, sustained proliferation, angiogenesis, invasion, and metastasis. On the other hand, ROS at elevated levels can increase the vulnerability of cancer cells to various inducers. Considering the dual nature of ROS and the complexity of tumors themselves, exploring approaches to rationally utilize CHM to regulate ROS may maximize the anticancer functions of CHM.

The first strategy is to exploit the antioxidant properties of CHMs to reduce excessive intracellular ROS and to antagonize ROS-induced protumorigenic effects on normal cells. However, many clinical trials have inconsistently concluded that antioxidant supplements are beneficial for preventing tumors; furthermore, the long-term use of certain antioxidant supplements may even increase the incidence of some tumors and overall mortality [3, 145]. Moreover, recent studies have shown that antioxidants can promote carcinoma proliferation and distant metastasis in vivo [6, 146]. In terms of cancer treatment, antioxidant supplements may reduce the side effects of chemo- and radiotherapy in some cases but may also antagonize the positive effects of these treatments [17, 147, 148]. Therefore, although the abovementioned antioxidant CHM compounds or their active ingredients have shown an initial positive effect in tumor prevention and have been shown as an adjuvant treatment in preclinical studies, caution must be taken in their long-term application. Antioxidant CHMs are different from antioxidant supplements due to the fact that they are natural products with a complex combination of active ingredients. The properties of antioxidant CHMs are closer to those of fruits and vegetables rich in antioxidants. Thus, the use of CHMs rich in antioxidants rather than a single antioxidant compound might have better effects in tumor prevention. However, further systematic studies are needed.

Compared to ROS reduction strategies, which have a controversial role in application to tumors, ROS promotion strategies have shown better anticancer effects and clinical prospects. Such strategies can be implemented by using an agent that either increases ROS production or reduces antioxidant capacity or results in a combination of both effects. Various chemotherapeutic drugs, molecular targeted drugs, radiotherapy, and photodynamic therapy have been shown to kill tumor cells by increasing intracellular ROS levels [56, 104, 149, 150]. To date, some novel ROS inducers (such as ARQ501 and elesclomol), as well as antioxidase system drugs (such as the SOD1 inhibitor ATN-224 and the GSH inhibitors buthionine sulfoximine (BSO) and phenethyl isothiocyanate (PEITC)), have also been under clinical trials (see http://clinicaltrials.gov/). Many CHM-derived active constituents act as ROS generators to exert anticancer effects. Importantly, the intracellular level of ROS should be carefully controlled when using ROS-generating CHMs. If the levels of ROS are not sufficiently increased to the toxicity threshold, downstream oncogenes, such as PI3K, HIFs, NF-*κ*B, and MAPK, may be activated to promote cancer development. Conversely, increasing the ROS levels too far over the cytotoxic level will lead to nonspecific damage to normal cells, thereby injuring sensitive vital organs such as the heart, liver, and kidneys [8]. Indeed, tumor cells maintain elevated antioxidant system activity to prevent oxidative damage from cytotoxic ROS; thus, ROS generators are not always useful. However, the use of antioxidant inhibitors in combination with ROS inducers may be a promising method in anticancer therapy because this approach can decrease the adaptability of tumor cells to both agents [20, 26]. Compounds such as curcumin [151] and triptolide [152] can simultaneously induce ROS generation and inhibit antioxidant defense, causing cancer cell death and enhancing the efficacy of chemotherapy. This pleiotropic effect may be beneficial in overcoming the resistance of cancer cells to conventional single-target drugs [149]. However, due to the bimodal nature of ROS and CHM, identifying the specific types of ROS and antioxidant molecules that are uniquely required for tumor growth and survival and determining the mechanisms targeted by the specific CHM in different types of tumors are important. Currently, the advent of new molecular tools for the localization, quantification, and real-time detection of ROS is expected to further deepen our understanding of redox, to advance ROS-based treatment strategies, and to generate great opportunities for the development of anticancer drugs from CHMs.

## 6. Conclusions

In summary, we describe how ROS are generated and eliminated within cells and the complicated dual roles of ROS in cancers. ROS not only are indiscriminate damaging molecules but also function as specific secondary messengers, involved in various physiological and pathological responses. This is the current focus on the debate in the field of redox biology and accounts for inconsistency with clinical and experimental studies on ROS. Traditional Chinese medicine is an ancient practice medicine with potential advantages in cancer treatment. We selected and summarized the original researches of CHM based on ROS regulation with relatively precise molecular mechanisms. CHMs exert antitumor effects through antioxidant activities, including inhibition of inflammation; prevention of carcinogenesis; inhibition of tumor proliferation, growth, and metastasis; and reduction of side effects of chemo- and radiotherapy; on the other hand, CHMs primarily induce multiple cell death to kill cancer cells selectively by promoting oxidation, cause DNA damage and enhance the efficacy of chemo/radiotherapy and molecular targeted agents, and reverse drug resistance of cancer cells. Taken together, CHM plays a vital role in the prevention and treatment of tumor initiation, development, and progression. Moreover, it is a promising strategy to develop low-toxic and effective antitumor agents from CHMs based on the regulation ROS. Notably, the majority of current mechanistic researches are based on the reductionist approach, which may not adequately clarify the efficacy of herbal medicines, especially for the traditional Chinese compound formulas, the most common way used in the clinic. Therefore, a systematic biological method may be more appropriate and efficient for the development of effective therapies; additionally, more well-designed clinical trials and transformational experimental studies are also vitally needed to confirm the efficacy of CHMs in humans.

## Figures and Tables

**Table 1:** Components of TCMs targeted to decrease ROS levels and the effects of these components in cells.

Components	Herbs	Target cells	Biological effects	Molecular events	Reference
Astragaloside IV	*Milkvetch root (Huang Qi)*	Kidney proximal tubular HK-2 cells	Mitigate cisplatin-induced acute kidney injury	T-SOD⬆, GSH-Px⬆, CAT⬆; KIM-1⬇, MDA⬇, TNF-*α*⬇; Nrf2⬆, HO-1⬆, NF-*κ*B⬇	[153]

Benzyl isothiocyanate	*Lepidii semen (Ting Li Zi)*	Leukemia HL-60 cells	Prevent inflammation-related carcinogenesis	NADPH oxidase⬇, ROS⬇	[154]

Catalpol	*Rehmannia glutinosa libosch (Di Huang)*	Pheochromocytoma PC-12 cells	Against LPS-induced apoptosis	Bcl-2⬆, BAX⬇, p-CaMK⬇, Ca2+⬇; CaMKII-dependent ASK-1/JNK/p38 pathway⬇	[155]

Crocin	*Crocus sativus L. (Zang Hong Hua)*	Melanoma B16F10 cells	Inhibition of melanogenesis	Tyrosinase⬇, MITF⬇, ROS⬇	[156]

Curcumin	*Curcuma longa L. (Jiang Huang)* *Curcumae radix (Yu Jin)*	Breast epithelial MCF-10A cells	Against PhIP-induced cytotoxicity	Nrf2⬆, FOXO⬆; BRCA-1, H2AFX, PARP-1, and P16⬆; Casp-3/9⬇	[157]

Dioscin	*Smilacis glabrae rhizoma (Tu Fu Ling)*	Ventricular H9c2 cells	Against doxorubicin-induced cardiotoxicity	miR-140-5p⬇; ROS, MDA, SOD, GSH, and GSH-Px⬇; Nrf2 and Sirt2 pathway⬆	[158]

Daphnetin	*Daphne Korean nakai (Chang Bai Rui Xiang)*	Monocyte RAW264.7 cells	Against t-BHP-triggered oxidative damage and mitochondrial dysfunction	ROS⬇, MDA⬇; SOD⬆, GSH/GSSG⬆; JNK and ER⬆; Nrf2/ARE pathway⬆	[159]

Ellagic acid	*Rubus idaeus (Fu Pen Zi)*	Ishikawa cells	Reduction of glycolytic flux Against doxorubicin-induced cardiac oxidative	ROS⬇, NHE1⬇, Na+/H+ exchanger activity⬇, PHi⬇; ROS⬇, MDA⬇, XO⬇; Casp-3, p-ERK1/2, p-p38, and NF-*κ*B⬇	[160] [161]

Eriodictyol	*Dracocephalum rupestre (Yan Qing Lan)*	Pheochromocytoma PC-12 cells	Against hydrogen peroxide-induced neurotoxicity	Nrf2, HO-1, *γ*-GCS, and GSH⬆; Nrf2/ARE pathway⬆	[162]

Epigallocatechin-3-gallate	*Green tea (Lv Cha)*	Inner ear UB/OC-1 cells	Against cisplatin-induced ototoxicity	ROS⬇, ERK1/2⬇, STAT3/STAT1⬆	[163]
		Breast epithelial MCF-10A cells	Against PhIP-induced breast carcinogenesis	Ras/ERK/Nox/ROS pathway⬇	[164]

Isoliquiritigenin	*Licorice (Gan Cao)*	Kidney epithelial LLC-PK1 cells	Against cisplatin-induced nephrotoxicity	ROS⬇, Casp-3⬇, Nrf2/HO-1⬆	[165]

Luteolin	*Chrysanthemi indici flos (Ye Ju Hua)* *Lonicerae japonicae flos (Jin Yin Hua)*	Bronchial epithelial BEAS-2B cells	Inhibition of Cr(VI)-induced carcinogenesis	AP-1, HIF-1*α*, COX-2, and iNOS⬇; MAPK, NF-*κ*B, and EGF⬇; proinflammatory cytokine⬇; Nrf2, HO-1, NADPH, and SOD1/SOD2⬆	[166, 167]

Nexrutine	*Phellodendron amurense (Huang Bai)*	Pancreatic cancer cells Capan-2, MIAPaCa-2, AsPC-1, BxPC-3	Inhibit autophagy and pancreatic cancer cell growth	ROS⬇, LC3-II⬇; STAT3⬇	[168]

Pedicularioside G	*Pedicularis striata (Ma Xian Hao)*	Hepatoma SMMC-7721 cells and HUVEC	Inhibition of angiogenesis and tumorigenesis	ROS⬇, VEGF⬇, IL-8⬇	[169]

Resveratrol	*Polygonum cuspidatum* *(Hu Zhang)* *Fructus mori* *(Sang Shen)*	Pancreatic stellate cells	Inhibition of invasion, migration, and glycolysis	ROS/miR-21⬇, PTEN⬆	[104]
		Glioblastoma U87 MG and GBM8401 cells	Enhance the efficacy of temozolomide	ROS/ERK-mediated autophagy⬇; apoptosis⬆	[170]

Rutin	*Ruta graveolens L. (Yun Xiang)* *Fagopyrum tataricum (L.) Gaertn (Ku Qiao Mai)*	Neuroblastoma IMR32 cells	Ameliorates doxorubicin-induced memory deficits Against cisplatin-induced nephrotoxicity	ROS/JNK/TNF/P38 MAPK pathway⬇	[171, 172]

Saikosaponin-D	*Radix bupleuri (Chai Hu)*	/	Reduces cisplatin-induced nephrotoxicity	ROS, P38, and JNK/NF-*κ*B pathway⬇	[173]

Sulforaphane	*Codonopsis radix (Dang Shen)*	Bronchial epithelial BEAS-2B cells	Against cadmium-induced carcinogenesis	Nrf2⬆, ROS⬇, protective autophagy⬆	[174]

Tetramethylpyrazine	*Chuanxiong rhizoma (Chuan Xiong)*	Kidney proximal tubular HK-2 cells	Against arsenite-induced nephrotoxicity	ROS⬇, GSH⬆, apoptosis⬇, proinflammatory signals⬇, cytotoxic autophagy⬇	[175]

Tanshinone II-A	*Radix salviae (Dan Shen)*	Monocyte RAW264.7 cells and stomach cancer MKN45 cells	Decrease *H. pylori*-induced inflammation and gastric cancer	NF-*κ*B and MAPK(p38/JNK) pathway⬇; inflammatory substance⬇; apoptotic protein⬆	[176]

**Table 2:** Compounds of TCMs targeted to increase ROS levels and the effects of these components in cancer cells.

Components	Herbs	Target cells	Biological effects	Molecular events	Reference
Aloe emodin	*Aloe (Lu Hui)*	Lung cancer cells H460	DNA damage Apoptosis	ROS⬆, SOD⬆; hMTH1, hOGG1, and APE⬇	[177]
		Nasopharyngeal cancer cells NPC-TW039, TW076	Cycle arrest	Cyclin B1⬆, Cdc2⬆, PARP⬆, Casp-3/8⬆	[178]

Atractyloside	*Atractylodes lancea (Cang Zhu)*	Leukemia cells	Apoptosis	ROS⬆; ER Ca2+⬆	[179]

Baicalein	*Scutellaria baicalensis (Huang Qin)*	Breast cancer cells ZR-75-1	Apoptosis	PLC-dependent Ca2+⬆, ROS⬆; Ca2+-associated apoptosis⬆	[180]
		Colon/prostate cancer cells SW480; PC3	Apoptosis/overcome TRAIL resistance	ROS⬆, DR5⬆, TRAIL receptor⬆	[181]

Berberine	*Coptis chinensis Franch (Huang Lian)*	Lung cancer cells H1975, H1650, H1819, A549, H1299	Apoptosis	SREBP1⬇; mitochondrial dysfunction, ROS⬆, p-AMPK⬆; lipogenesis⬇	[182]
		Liver cancer cells HepG2, Hepa1-6	Apoptosis	p-PTEN, p-Akt, p-mTOR, and p-PDK1⬇; FoxO1, FoxO3, Bim, Bax, Bax/Bcl-2, Casp-3/9, and cl-PARP⬆; p-JNK⬆, ROS⬆; SOD, CAT, and GSH⬇	[183]

Bufalin	*Bufo bufo gargarizans cantor (Chan Su)*	Breast cancer cells MCF-7, MDA-MB-231	Necroptosis	RIP1/RIP3⬆; ROS and PARP-1⬆, RIP1/RIP3/PARP-1 pathway⬆	[184]
		Colon cancer cells HT-29, Caco-2	Autophagy	ROS⬆, p-JNK⬆; ATG5, Beclin-1, LC3-II, and autophagic flux⬆	[185]

Celastrol	*Tripterygii radix (Lei Gong Teng)*	Osteosarcoma cells HOS, MG-63, U-2OS, Saos-2	Apoptosis Autophagy Cycle arrest	ROS/JNK pathway⬆, Casp-3/8/9⬆; LC3-II⬆	[186]
		Breast cancer cells MCF-7, MCF-7/MDR	Against doxorubicin resistance	HSF-1⬆, NF-*κ*B⬇, P-gp⬇	[187]

Cordycepin	*Cordyceps sinensis (Dong Chong Xia Cao)*	Glioma cells Rat C6, LN18, T98G, LN229, SHG-44	Apoptosis Cycle arrest Synergistic with TMZ	ROS⬆, GSH⬇; p-GSK-3*β*⬇, *β*-catenin⬇	[188]
		Gastric/colon cancer cells SGC-7901; HT-29	DNA damage Apoptosis	DR3⬆, A3AR⬇, PI3K/Akt⬆, ROS⬆, *Δψ*m⬇; p53, Bax, Casp-3/8/1, and cl-PARP⬆	[189, 190]

Costunolide	*Aucklandiae radix (Mu Xiang)*	Prostate cancer cells PC-3, DU-145	Enhance doxorubicin-induced apoptosis	ROS⬆, p-JNK⬆, p-p38⬆, *Δψ*m⬇, Bax⬆, Bak⬆, Bcl-2⬇, Bcl-xL⬇, Casp-3/9⬆, cl-PARP⬆	[191]
		Ovarian cancer cells MPSC1, A2780, SKOV3	Induce apoptosis of platinum-resistant cells Apoptosis	ROS⬆, Bcl-2⬇, Casp-3/8/9⬆	[192]
		Colon cancer cells HCT116		TrxR1⬇, ROS⬆, ERS⬆	[193]

Cucurbitacin E	*Bolbostemae paniculati bulbus (Tu Bei Mu)*	Colorectal cancer primary cell lines	Cycle arrest Apoptosis	ROS⬆, *ΔΨ*m⬇, GADD45*γ*⬆, Cdc2⬇ Cyclin B1⬇	[194]

Curcumin	*Curcuma longa L. (Jiang Huang)*	Lung/prostate cancer cells A549, PC-3	Apoptosis	TrxR2⬇; Bax/Bcl-2⬆, *Δψ*m⬇, cyto C⬆, Casp-3/9⬆	[195]
		Cervical cancer cells C33A, CaSki, HeLa, ME180		p-PERK, IRE-1*α*,GRP-78, ATF6, and CHOP⬆; Bax/Bcl-2, Casp-3/9, and cl-PARP⬆	[196]

Daidzein	*Glycine max (Hei Dou)*	Breast cancer cells MCF-7	Apoptosis	ROS⬆, *ΔΨ*m⬇, Bcl-2⬇, Bax⬆, cyto C⬆, Casp-7/9⬆	[197]

Emodin	*Rheum palmatum (Da Huang)*	Cervical cancer cells HeLa	Apoptosis	ROS⬆, NF-*κ*B⬇, AP-1⬇, p-P38⬆	[198, 199]

Epigallocatechin-3-gallate	*Green tea (Lv Cha)*	Malignant B-cell lines HS-sultan, RPMI8226	Apoptosis	Cyto C, Smac/DIABLO, AIF, and Casp-3/9⬆	[200]

Escin	*Semen aesculi (Suo Luo Zi)*	Osteosarcoma cells MNNG/HOS, Saos-2, MG-63, U2-OS	Autophagy Apoptosis	ROS/p38 MAPK⬆; LC3 II, ATG5 ATG12 and Beclin⬆; Bax/Bcl-2⬆, Casp-3/7/8/9⬆	[201]

Eugenol	*Eugenia caryophyllata (Ding Xiang)*	Leukemia cells HL-60, U937	Apoptosis	ROS⬆, *ΔΨ*m⬇, Bcl-2⬇, cyto C⬆ Casp-3/9⬆	[202]

Evodiamine	*Evodia rutaecarpa Bentham (Wu Zhu Yu)*	Glioma cells U87-MG	Apoptosis Autophagy Cycle arrest	Calcium/JNK signaling-mediated autophagy⬆; calcium/mitochondria-mediated apoptosis⬆	[203]
		Cervical cancer cells HeLa		PTK/Ras-Raf-JNK⬆; ROS/NO⬆; p53, p21, Cdc2, and cyclin B1⬆	[204]

Gambogic acid	*Garcinia hanburyi Hook. f. (Teng Huang)*	Colon cancer cells HCT-15, HCT-15R	Apoptosis/against drug resistance	*ΔΨ*m⬇, cyto C⬆, AIF⬆; Bcl-2, Bcl-xl, Mcl-1, XIAP and survin⬇; p-JNK⬆, c-JUN⬆;	[205]
		Lung cancer cells A549,H460,H1299	Synergistic with cisplatin	Casp-3/8/9, Fas and Bax⬆; Bcl-2,XIAP, survivin⬇; NF-*κ*B, MAPK/HO-1 pathway⬇	[206]

Germacrone	*Curcuma zedoaria (E Zhu)*	Breast cancer cells MCF-7,MDA-MB-231	Apoptosis	ROS⬆, *ΔΨ*m⬇; Bax, JNK1, IKK*α*, and IKK*β*⬆; Bcl-2, Bcl-xl, Bim, and Bik⬇	[207]

Gypenoside	*Gynostemma pentaphyllum (Jiao Gu Lan)*	Esophageal cancer cells ECA-109, TE-1	Autophagy	ROS-induced ERS⬆, Ca2+⬆; P62⬆, autophagic flux⬇	[208]

Honokiol	*Magnolia officinalis (Hou Po)*	Osteosarcoma/glioma cells HOS, U2OS; U87MG Neuroblastoma cells Neuro-2a, NB41A3	Apoptosis Autophagy Cycle arrest	GRP-78⬆, ROS⬆, p-ERK1/2⬆; LC3 II⬆ ERS/ROS/ERK1/2 pathway⬆; p53/PI3K/Akt/mTOR⬇	[209–211]

Icariin	*Epimedium brevicornum Maxim (Yin Yang Huo)*	Esophageal cancer cells EC109, TE1 Liver cancer cells SMMC-7721, Bel-7402, L-02	Apoptosis	GSH⬇; NADPH, Casp-9⬆; p-PERK, GRP-78, ATF4, p-eIF2*α*, and CHOP⬆; PUMA⬆, Bax/Bcl-2⬆, *ΔΨ*m⬇, cyto C⬆, Casp-3/9⬆, cl-PARP⬆; p-JNK⬆	[212, 213]

Isoalantolactone	*Inula helenium (Tu Mu Xiang)*	Esophageal cancer cells ECA109, EC9706, TE-1, TE-13	Apoptosis	ROS⬆, DR5⬆, Casp-3/7/10⬆; DR5-induced extrinsic apoptosis	[214]

Luteolin	*Chrysanthemi indici flos (Ye Ju Hua)* *Lonicerae japonicae flos (Jin Yin Hua)*	Liver cancer cells HepG2; MDR cancer cells	Apoptosis DNA damage Cycle arrest	ROS⬆, PIG3⬆, *ΔΨ*m⬇, cyto C⬆, Bax/Bcl-2⬆, casp-3/9⬆ ROS⬆, ATR/Chk2/p53 pathway⬆, p38⬆, Bcl-2⬇, NF-*κ*B⬇	[215, 216]

Matrine	*Sophora flavescens (Ku Shen)*	Pancreatic cancer cells PANC-1, Miapaca-2	Cycle arrest Apoptosis	ROS, p-ERK, p-JNK, and p-P38⬆ Cyclin A, D1, CDK2⬇; cyto C, Casp-3, cl-PARP, Bax, and Bad⬆; Bcl-2⬇	[217]
		Liver cancer cells HepG2	Program cell death	*ΔΨ*m⬇, ROS⬆; Fas⬆, Fas-L⬆; Casp-3⬆; AIF translocation⬆	[218]

Neferine	*Nelumbo nucifera (Lian Hua)*	Lung cancer cells A549	Autophagy enhances cisplatin-induced autophagic cell death	ROS⬆, GSH⬇, PI3K/Akt/mTOR⬇, LC3-II⬆ PI3K/Akt/mTOR pathway⬇	[219, 220]

Norcantharidin	*Mylabris (Ban Mao)*	Liver cancer cells HepG2	Apoptosis	ROS⬆, *ΔΨ*m⬇, cyto C⬆, Bcl-2⬇ Bax, Casp-3/9, and cl-PARP⬆	[221]

Oridonin	*Rabdosia rubescens (Dong Ling Cao)*	Osteosarcoma cells MG-63, HOS	Apoptosis	PPAR-*γ*⬆, Nrf2⬇; *ΔΨ*m⬇ Bax/Bcl-2⬆, Casp-3/9⬆	[222]

Oroxylin A	*Oroxylum indicum (Mu Hu Die)* *Scutellaria baicalensis (Huang qin)*	Colon cancer cells CaCo-2, HCT-116	Apoptosis	UCP2⬇; ROS, MPTP, cyto C, AIF Casp-3/9 and cl-PARP⬆	[223]

Plumbagin	*Plumbago zeylanica (Bai Hua Dan)*	Colon cancer cells HT-29, HCT-116, Caco-2	Apoptosis	ASK1/TRAF2⬆, JNK⬆; mTORC1⬇ Bcl-2⬇	[224]

Resveratrol	*Polygonum cuspidatum (Hu Zhang)* *Fructus Mori (Sang Shen)*	Lung/breast cancer cells H1299, MCF-7	Apoptosis Autophagy	TIGAR⬇, GSH⬇, ROS⬆, cl-PARP⬆ LC3-II⬆	[225]
		Prostate cancer cells LNCaP, PC-3	Apoptosis	TRX1⬇, TXNIP⬆	[226]

Saxifragifolin D	*Androsace umbellate (Hu Er Cao)*	Breast cancer cells MCF-7, MDA-MB-231	Autophagy Apoptosis	LC3-II, Beclin-1, and Vps34⬆; ROS-mediated ERS⬆	[227]

Sophoranone	*Radix sophorae tonkinensis (Shan Dou Gen)*	Leukemia cells U937 cell	Apoptosis	ROS⬆, MPTP⬆, cyto C⬆, p-JNK⬆, Casp-3⬆	[228]

Tetrandrine	*Stephania tetrandra radix (Fang Ji)*	Leukemia cells K562, CMK, HEL	Autophagy Cycle arrest Differentiation	ROS⬆, LC3 II⬆, p-Akt⬆; p21⬆, p27⬆	[229]
		Live cancer cells Huh7, HepG2, BEL7402	Apoptosis	ROS⬆; p-Akt, p-ERK1/2, and p-JNK⬇	[230]

Vitexin	*Vitex negundo L (Ma Bian Cao)*	Melanoma cells A375, Sk-Mel-5, Sk-Mel-28	DNA damage Cycle arrest	ROS, Bax, and PARP⬆; Bcl-2⬇ p-ATM, p-ATR, p-CHK2, p53, p21, and *γ*-H2AX⬆	[231]
